# Ahnak in the prefrontal cortex mediates behavioral correlates of stress resilience and rapid antidepressant action in mice

**DOI:** 10.3389/fnmol.2024.1350716

**Published:** 2024-05-17

**Authors:** Dionnet L. Bhatti, Junghee Jin, Jia Cheng, Kathryn McCabe, Ko-Woon Lee, Clara Berdasco, Yu Young Jeong, Subhash C. Sinha, Yong Kim

**Affiliations:** ^1^Laboratory of Molecular and Cellular Neuroscience, The Rockefeller University, New York, NY, United States; ^2^Program in Neuroscience, Harvard Medical School, Boston, MA, United States; ^3^Department of Neurosurgery, Robert Wood Johnson Medical School, Rutgers University, Piscataway, NJ, United States; ^4^Weill Cornell Medicine Helen & Robert Appel Alzheimer’s Disease Research Institute, New York, NY, United States; ^5^Brain Health Institute, Rutgers University, Piscataway, NJ, United States

**Keywords:** chronic stress, susceptibility, resilience, prefrontal cortex, Ahnak, ketamine, S100a10, Anxa2

## Abstract

The prefrontal cortex (PFC) is a key neural node mediating behavioral responses to stress and the actions of ketamine, a fast-acting antidepressant. The molecular mechanisms underlying these processes, however, are not fully understood. Our recent study revealed a pivotal role of hippocampal Ahnak as a regulator of cellular and behavioral adaptations to chronic stress. However, despite its significant expression in the PFC, the contribution of cortical Ahnak to behavioral responses to stress and antidepressants remains unknown. Here, using a mouse model for chronic social stress, we find that Ahnak expression in the PFC is significantly increased in stress-resilient mice and positively correlated with social interaction after stress exposure. Conditional deletion of Ahnak in the PFC or forebrain glutamatergic neurons facilitates stress susceptibility, suggesting that Ahnak is required for behavioral resilience. Further supporting this notion, Ahnak expression in the PFC is increased after the administration of ketamine or its metabolite (*2R*, *6R*)-hydroxynorketamine (HNK). Moreover, Ahnak deletion in forebrain glutamatergic neurons blocks the restorative behavioral effects of ketamine or HNK in stress-susceptible mice. This forebrain excitatory neuron-specific Ahnak deletion reduces the frequency of mini excitatory postsynaptic currents in layer II/III pyramidal neurons, suggesting that Ahnak may induce its behavioral effects via modulation of glutamatergic transmission in the PFC. Altogether, these data suggest that Ahnak in glutamatergic PFC neurons may be critical for behavioral resilience and antidepressant actions of ketamine or HNK in chronic social stress-exposed mice.

## Introduction

Stress exposure can elicit adaptive changes in molecular expression and neural circuit activity that promote survival in the face of danger. Repeated exposure to unavoidable stress, however, often results in maladaptive molecular and cellular processes that may, in turn, contribute to the etiology of neuropsychiatric disorders including major depressive disorder (MDD) (Kessler, 1997; Kendler et al., 1999; Nestler et al., 2002; McEwen, 2012; Syed and Nemeroff, 2017). While stress exposure can render some individuals who are susceptible to developing MDD, others are resilient and often remain healthy (Southwick et al., 2005). Several animal models, including ‘learned helplessness’, chronic unpredictable stress, restraint stress, and chronic social defeat stress (CSDS), have been used to uncover key cellular and molecular mechanisms mediating stress susceptibility or resilience (Cryan and Mombereau, 2004; Krishnan et al., 2007; Nasca et al., 2015). Among them, studies using the mouse CSDS paradigm have revealed many molecular, cellular, and neural circuit mechanisms underlying individual divergence in response to chronic stress (Han and Nestler, 2017; Cathomas et al., 2019).

In a previous study, we adopted the CSDS paradigm to examine alterations in molecular expression and neuronal activity in the ventral hippocampus after chronic stress (Bhatti et al., 2022). Among four different neuronal types in the ventral dentate gyrus, the firing of parvalbumin-expressing interneurons (PV neurons) is significantly increased in stress-susceptible mice compared with that of stress-resilient mice or non-defeat control mice. Furthermore, we found that this increased PV neuronal activity in the ventral dentate gyrus is required for CSDS-induced social avoidance (Bhatti et al., 2022). We also found that Ahnak is one of the altered molecules in PV neurons in response to CSDS, and it mediates behavioral adaptation in response to CSDS (Bhatti et al., 2022).

Ahnak is a major binding partner and protein stabilizer of S100a10 (also called p11) and annexin A2 (Anxa2) (Jin et al., 2020). Alterations of p11 have been implicated in the etiology of major depressive disorder and antidepressant actions (Svenningsson et al., 2006; Alexander et al., 2010; Svenningsson et al., 2013; Chen et al., 2022). p11 and Anxa2 form a heterotetramer complex (Benaud et al., 2004; De Seranno et al., 2006; Rezvanpour et al., 2011; Oh et al., 2013). A peptide motif present in the C-terminal region of Ahnak binds to the p11/Anxa2 heterotetramer (Oh et al., 2013). Ahnak also serves as a regulator of L-type voltage-gated calcium channels in neurons (Jin et al., 2020), cardiomyocytes (Haase et al., 2005), and T cells (Matza et al., 2008). Genetic polymorphism of L-type α1 pore-forming subunit and axillary *β* subunits of voltage-gated calcium channels is highly associated with major psychiatric disorders including MDD (Green et al., 2010; Cross-Disorder Group of the Psychiatric Genomics, 2013; Schizophrenia Working Group of the Psychiatric Genomics, 2014; Pinggera et al., 2015). Thus, alterations in Ahnak expression and its function might underlie the pathophysiology of psychiatric disorders or contribute to the actions of antidepressants.

While Ahnak deletion selectively in PV neurons renders antidepressant-like behavioral outcomes, constitutive or forebrain glutamatergic neuron-specific Ahnak deletion in mice induces depression-like phenotypes, including reduced reward seeking and active coping behaviors, similar to that of constitutive p11 KO mice (Jin et al., 2020). Among regions within the glutamatergic forebrain, the PFC has been implicated in the mechanisms by which p11 regulates chronic stress-induced depression-like behavior (Seo et al., 2017). The PFC has also been proposed as a critical neural node for the actions of fast-acting antidepressants, such as ketamine (Fuchikami et al., 2015; Ghosal et al., 2020). However, the role of Ahnak in glutamatergic forebrain neurons in the PFC for behavioral responses to CSDS and ketamine has not been investigated. In this study, we examine Ahnak expression in the PFC in response to chronic stress and harness cell-type and region-specific Ahnak deletions to examine a role of cortical Ahnak in chronic stress-induced behavioral responses and the rapid antidepressant-like effects of ketamine. Although an overexpression approach would be beneficial, overexpression of Ahnak is not technically feasible due to its large size (~680 kDa) (Hieda et al., 1989; Shtivelman et al., 1992).

## Materials and methods

### Animals

All experiments involving animals were approved by The Rockefeller University Institutional Animal Care and Use Committee and were in accordance with the National Institutes of Health guidelines. Constitutive Ahnak knockout (KO) and Floxed Ahnak mice were generated and maintained at The Rockefeller University as described previously (Jin et al., 2020). Floxed Ahnak mice were crossed with EMX-Cre (stock 005628, The Jackson Laboratory) to generate forebrain glutamatergic neuron-specific Ahnak KO line. The crossings of floxed Ahnak with EMX-Cre were assisted by *in vitro* fertilization (IVF) and embryo transfer techniques (Transgenic and Reproductive Technology Center, The Rockefeller University), which provide genotype- and age-matched animals. All mice are male of C57BL/6 background, except for CD1 aggressors (strain 022, Charles River, Kingston, NY), used for CSDS. Mice were housed 3–5 per cage with a 12:12 h light/dark cycle and *ad libitum* access to food and water. All behavioral tests were performed during the light cycle. All behavioral experiments with the CSDS paradigm commenced with male mice aged 8–12 weeks old. In addition, ~ 4-month-old constitutive Ahnak KO and WT controls were used for the test of ketamine and fluoxetine. Transgenic mice were assigned randomly to experimental stress conditions based on their genotype. For AAV-mediated gene delivery experiments, transgenic mice were randomly assigned to experimental virus groups and then experimental stress conditions. Mice from each group were evenly assigned to equipment and run in parallel during behavioral tests.

### Stereotaxic surgery

All stereotaxic surgeries were performed on an Angle Two Small Animal Stereotaxic Instrument (Leica Biosystems, Buffalo Grove, IL) with a microinjection syringe pump (UMP3 UltraMicroPump, World Precision Instruments, Sarasota, FL), as described previously (Bhatti et al., 2022). Male mice (7–8 weeks of age) were anesthetized with a mix of ketamine (100 mg/mL) and xylazine (10 mg/mL). AAV5-hSyn-GFP and AAV5-hSyn-Cre-GFP were obtained from UNC Vector Core. Viruses (300 nL/side) were injected bilaterally into the PFC (AP + 1.6 ML + 0.4 DV-2.3 mm relative to bregma) with a 2 mL Hamilton Neuros syringe at a speed of 0.1 μL/min, targeting primarily prelimbic cortex. The needle was left for an additional 10 min and then slowly withdrawn. Mice were monitored for 48 h to ensure full recovery from the surgery. Experiments commenced 3 weeks after stereotaxic surgery to allow optimal expression of AAVs.

### Chronic social defeat stress

Chronic social defeat stress (CSDS) was carried out as described previously (Golden et al., 2011; Bhatti et al., 2022). Retired male breeder CD-1 mice were screened for 3 consecutive days, and aggressors were selected according to the following criteria: (i) the latency to the initial attack was under 60 s and (ii) the screener mouse was attacked for 2 consecutive days. For 10 consecutive days, the experimental mice were placed in the home cage of a prescreened CD-1 aggressor for 5 min of physical attack and then separated by a perforated divider for the remaining 24 h until the next defeat of 24 h. Each experimental mouse was exposed to a different aggressor each day. In parallel, stress-naïve control mice were placed in pairs within an identical home cage setup separated by a perforated divider for the duration of the defeat sessions. They were never in physical or sensory contact with CD-1 mice. After 10 days of social defeat, all aggressors and experimental mice were separated and singly housed. The social interaction (SI) test was conducted after 24 h.

### Subthreshold social defeat stress

Subthreshold social defeat stress (SSDS) was carried out as described previously (Cheng et al., 2019). Experimental mice were placed in the home cage of a prescreened CD-1 aggressor for 5 min of physical attack. After 15 min, the experimental mice were introduced to another 5 min of physical attack by a novel CD-1 aggressor. This procedure was repeated once more for three defeat sessions. The SI test was performed 24 h later.

### Social interaction test

Social interaction (SI) test was conducted as described previously (Golden et al., 2011). The test was composed of two phases, each consisting of 150 s, where the experimental mice were allowed to explore an open field (42 cm × 42 cm × 42 cm) with a wire mesh enclosure (10 cm wide × 6.5 cm deep × 42 cm high). In the first phase, the wire mesh enclosure was empty. In the second phase, a novel CD-1 aggressor mouse was placed inside the wire mesh. The amount of time the experimental mice spent in the interaction zone surrounding the wire mesh enclosure was collected and analyzed by the video-tracking apparatus and software EthoVision XT 7 (Noldus Information Technology, Leesburg, VA). The SI ratio was calculated by dividing the amount of time the experimental mice spent in the interaction zone in phase two by the time in phase one [SI Ratio = (phase 2 time)/(phase 1 time)]. Susceptible mice were defined by an SI ratio under 1, whereas resilient mice were defined by an SI ratio greater than 1 (Golden et al., 2011).

### Sucrose preference test

Sucrose preference test (SPT) was adapted from previous studies (Jin et al., 2020). During the 1-day habituation period, mice were given a choice of two water bottles. The following day, bottles were replaced with new bottles containing either water or 2% sucrose solution. The consumption of water and sucrose solution was measured after 24 h. The sucrose preference was represented as percent preference for sucrose [(sucrose consumed/(water+sucrose consumed)) × 100].

### Novelty suppressed feeding test

Novelty suppressed feeding (NSF) test was conducted as described previously (Lee et al., 2015). The Ahnak KO or WT mice were given saline (vehicle), ketamine (10 mg/kg, i.p.) or fluoxetine (15 mg/kg, i.p., daily for 3 weeks) and then subjected to food deprivation for 24 h in the home cage (after the last injection in case of fluoxetine). The mice were habituated in the test room for 30 min. After placing the mice in the corner of the test box (42 × 42 × 30.5 cm), the latency to eat a food pellet located in the center of the test box was measured. After the test, the mice were returned to their home cage and allowed to eat food pellets for 10 min, and food consumption was measured for home cage feeding.

### Forced swim test and tail suspension test

Forced swim test (FST) and tail suspension test (TST) were conducted as described previously (Lee et al., 2015). We used an automated TST/FST device (Clever Sys, Reston, VA, USA) for measuring the duration of behavioral immobility. For the TST, the mice were suspended by the tail and video-recorded for 6 min. Immobility during the last 4 min was analyzed. For FST, the mice were placed in a glass cylinder (15 cm diameter, 35 cm height), which was filled with water to a height of 15 cm (22–24°C), and video-recorded for 6 min. Immobility time during the last 4 min was analyzed.

### Drug administration

WT and Ahnak KO mice were given chronic fluoxetine (15 mg/kg, i.p., daily for 3 weeks) or a single injection of ketamine (10 mg/kg, i.p., Sigma–Aldrich). After 24 h of the last injection, the first behavioral test (NSF) was conducted. In the CSDS experiment, susceptible mice were identified based on the SI test. Ketamine administration (10 mg/kg, i.p.) or (2R, 6R)-hydroxynorketamine (HNK, 10 mg/kg, i.p.) were used. Fluoxetine, ketamine, or HNK were dissolved in saline (0.9% sodium chloride), and saline was used as a vehicle.

### Synthesis of (2R, 6R)-HNK

(*2R, 6R*)-HNK was prepared using racemic NK (norketamine) as described in the study conducted by Zanos et al., 2016. Commercially available racemic NK (norketamine) was resolved using (*L*)-(*+*)-tartaric acid, and the resulting enantiomerically pure (*R*)-NK. tartrate salt was basified by Aq. NaOH and concomitantly Boc-protected using Boc_2_O under the basic condition. Resulting N-Boc-protected (R)-NK was converted into TMS-enol ether using LDA and TMSCl treatment, and the product was epoxidized using mCPBA and treated with TBAF giving N-Boc-protected (*2R, 6R*)-HNK. The latter product was N-Boc deprotected using TFA, and resulting (*2R, 6R*)-HNK.TFA salt was basified using Aq. NaHCO_3_. Subsequently, the mixture was extracted using EtOAc and precipitated using 4 M HCl in dioxane to afford the (*2R, 6R*)-HNK.HCl salt. Identity of all products was determined using spectral (^1^HNMR and MS) analysis and compared with the reported data (Zanos et al., 2016). Purity and enantiomeric purity were determined using HPLC and chiral HPLC (chiralpak AD column, eluants: 60% EtOH in hexanes) (Zanos et al., 2016).

### Slice electrophysiology

Mice were euthanized with CO_2_. After decapitation and removal of the brains, transversal slices (300 μm thickness) were cut using a Vibratome 1,000 Plus (Leica Microsystems, USA) in cold NMDG-containing cutting solution (in mM): 105 NMDG (N-Methyl-D-glucamine), 105 HCl, 2.5 KCl, 1.2 NaH2PO4, 26 NaHCO3, 25 Glucose, 10 MgSO4, 0.5 CaCl2, 5 L-ascorbic acid, 3 sodium pyruvate, and 2 thiourea (pH was approximately 7.4, with osmolarity of 295–305 mOsm). After cutting, slices were left to recover for 15 min in the same cutting solution at 35°C and for 1 h at room temperature (RT) in recording solution (see below). Whole-cell patch-clamp recordings were performed with a Multiclamp 700B/Digidata1550A system (Molecular Devices, Sunnyvale CA, USA). The extracellular solution used for all recordings contained (in mM): 125 NaCl, 25 NaHCO3, 2.5 KCl, 1.25 NaH2PO4, 2 CaCl2, 1 MgCl2, and 25 glucose (bubbled with 95% O_2_ and 5% CO_2_). The slice was placed in a recording chamber (RC-27 L, Warner Instruments, USA) and constantly perfused with oxygenated CSF at room temperature.

For measuring the action potential firing, we used whole-cell current-clamp recordings. The intracellular solution contained (in mM): 126 K-gluconate, 4 NaCl, 1 MgSO4, 0.02 CaCl2, 0.1 BAPTA, 15 glucose, 5 HEPES, 3 ATP, and 0.1 GTP (pH 7.3). Small currents were injected to the cells to bring the membrane potential at −70 mV. Consecutive current steps of 25 pA were injected to induce depolarization. The frequency of action potentials was measured using the first two action potentials evoked by the respective injected current. Action potential properties were measured from the first action potential to avoid any confounding effects of adaptation.

For measuring mini excitatory postsynaptic currents (mEPSC), TTX (0.5 mM) and bicuculline (10 mM) were added to the recording solution to block action potentials and GABA receptors, respectively. Cell membrane potential was measured at −70 mV.

Data were acquired at a sampling frequency of 100 kHz, filtered at 1 kHz, and analyzed offline using pClamp10 software (Molecular Devices, Sunnyvale, CA, USA). All electrophysiological data are expressed as means ± SEM. Statistical analysis was conducted by Student’s *t*-test using GraphPad Prism 5. In all experiments, *p* < 0.05 was considered significant.

### Western blot

Mouse PFC tissues were lysed with a lysis buffer (Pierce IP Lysis Buffer, Thermo Fisher Scientific) supplemented with a protease and phosphatase inhibitor cocktail (78,442, Thermo Fisher Scientific). The tissue lysates were homogenized with a Tissue Grinder (10 strokes, 02–911-529, Thermo Fisher Scientific) and centrifuged at 800 × *g* for 5 min. Protein levels in the supernatant were measured by the BCA method. The samples were mixed with the standard protein sample buffer and boiled on a hot plate for 2 min and subjected to SDS-PAGE with 4–20% Novex Tris-Glycine gels (Thermo Fisher Scientific), followed by protein transfer onto a nitrocellulose membrane. The membranes were immunoblotted with incubation of anti-Ahnak (rabbit polyclonal, RU2064, 1:5,000) or anti-Gapdh (mouse monoclonal, MAB374, Millipore, 1:5,000) primary antibody followed by incubation of horseradish peroxidase-linked goat anti-rabbit or anti-mouse secondary antibody (1:5,000, Thermo Fisher Scientific). Antibody binding was detected using the enhanced chemiluminescence immunoblotting detection system (Perkin Elmer LLC WESTERN LIGHTNING PLUS-ECL, Thermo Fisher Scientific) and the Kodak autoradiography film. The bands were quantified by densitometry using NIH Image 1.63 software.

### Statistics

All data are expressed as means ± SEM except pie charts and correlation graphs. Sample sizes for biochemistry, electrophysiology, and CSDS were determined based on our empirical data accumulated in the laboratory and previous studies using CSDS (Berton et al., 2006; Krishnan et al., 2007; Menard et al., 2017). Sample sizes and statistical methods are provided in each figure legend. The statistical analysis was conducted using two-tailed unpaired Student’s *t*-test, one-way ANOVA, two-way ANOVA, or repeated-measures two-way ANOVA. ANOVA of paired Gaussian distributions was followed with Bonferroni’s or Newman–Keuls *post-hoc* test. The D’Agostino–Pearson omnibus normality test was used to determine normality. GraphPad Prism was used for statistical analysis and graphical preparations. *p* < 0.05 was considered the cutoff for statistical significance. Statistical significance is shown as *p < 0.05, **p < 0.01, ***p < 0.001, and ****p < 0.0001, otherwise indicated in figures or figure legends.

## Results

### High expression levels of Ahnak in the PFC are correlated with behavioral resilience to chronic social stress

In this study, we used the mouse CSDS paradigm, in which mice are subjected to repeated exposure of ethologically relevant stressors such as physical defeat and sensory contact with aggressive conspecifics to elicit social avoidance and anhedonic-like behavior (Krishnan et al., 2007; Golden et al., 2011). These behavioral phenotypes are determined by decreased time in social interaction (SI) with a novel aggressor and the amount of sucrose reward consumed in the home cage, respectively. Notably, an SI ratio comprising of the ratio of time spent in the interaction zone in the presence or the absence of a novel aggressor allows experimenters to divide cohorts on mice based on stress-induced behavioral outcomes. Specifically, using this SI ratio after CSDS, a proportion of mice is deemed as stress-susceptible (SI ratio < 1) or stress-resilient (SI ratio ≥ 1) (Krishnan et al., 2007; Golden et al., 2011).

**Figure 1:**
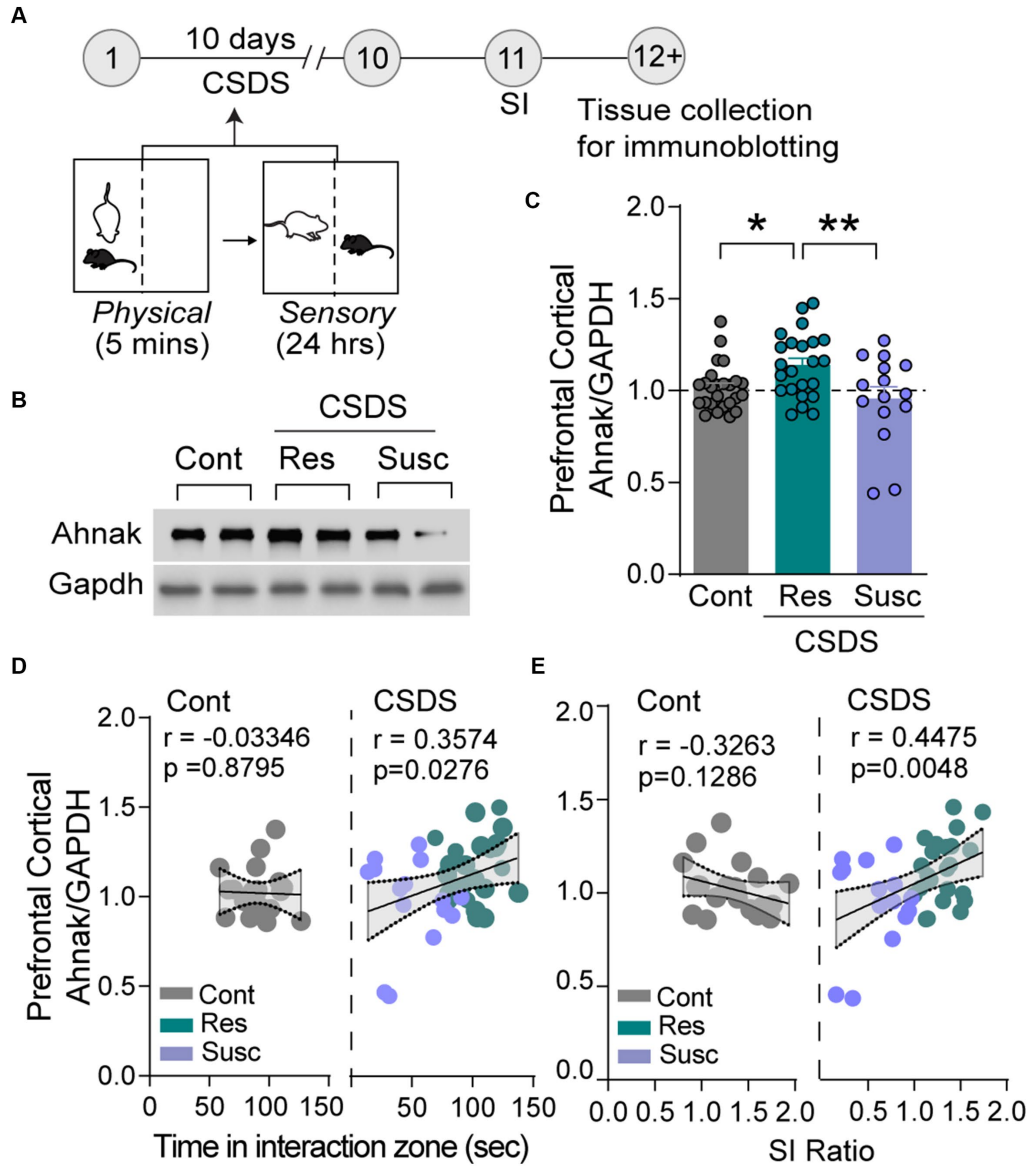
Stress resilience is associated with increased Ahnak expression in prefrontal cortex. **(A)** Diagram and timeline of CSDS and tissue collection. **(B)** Representative images of immunoblotting of Ahnak and Gapdh as a control. **(C)** Quantification of immunoblots. Non-defeated (Cont, *n* = 23 mice), resilient (Res, SI ratio ≥ 1, *n* = 23 mice), and susceptible (Sus, SI ratio < 1, *n* = 15 mice). *p < 0.05, **p < 0.01, one-way ANOVA with Tukey’s *post-hoc* comparison. **(D)** Ahnak protein expression in the PFC of mice exposed to CSDS (right, Pearson *r* = 0.3574, *p* = 0.0276, *n* = 38), but not no-defeat controls (left, Pearson *r* = −0.0335, *p* = 0.8795, *n* = 23 mice), is positively correlated with time in the interaction zone (with a caged aggressor) and **(E)** SI ratio (right, Pearson *r* = 0.4475, *p* = 0.0048, *n* = 38) but not non-defeated controls (left, Pearson *r* = −0.3263, *p* = 0.1286, *n* = 23 mice). All data were normalized to the Cont group.

Ahnak is expressed in the layer II/III of the PFC (Jin et al., 2020). First, we sought to examine whether behavioral outcomes after CSDS were associated with changes in its expression. After CSDS and SI, the PFC tissue was collected from stress-resilient, stress-susceptible, and non-defeated control mice to assess protein expression using immunoblotting (Figure 1A). Ahnak protein levels were significantly higher in the PFC of resilient mice compared with the levels in susceptible mice or non-defeated control mice (Figures 1B,C). Ahnak expression in the PFC of mice exposed to CSDS was also positively correlated with time spent in the interaction zone containing an aggressive conspecific (Figure 1D) and SI ratio (Figure 1E), suggesting that Ahnak may play a role in CSDS-induced behavioral adaptations.

### Ahnak in glutamatergic neurons in the PFC is required for behavioral resilience to chronic social stress

To assess whether Ahnak in the PFC is required for behavioral responses to chronic stress, we first examined whether selective deletion of Ahnak in the PFC would alter the behavioral consequence of CSDS. To achieve conditional deletion of Ahnak in a region-specific manner, we injected adeno-associated virus (AAV) expressing Cre recombinase fused to GFP, or empty vector GFP as a control, into the PFC of floxed Ahnak mice (cKO^PFC^). After 3 weeks, mice were subjected to CSDS (Supplementary Figure S1A). We found that conditional deletion of Ahnak in the PFC resulted in higher proportion of stress-susceptible compared with control (Supplementary Figures S1B–D) as indicated by increased social avoidance after CSDS.

**Figure 2:**
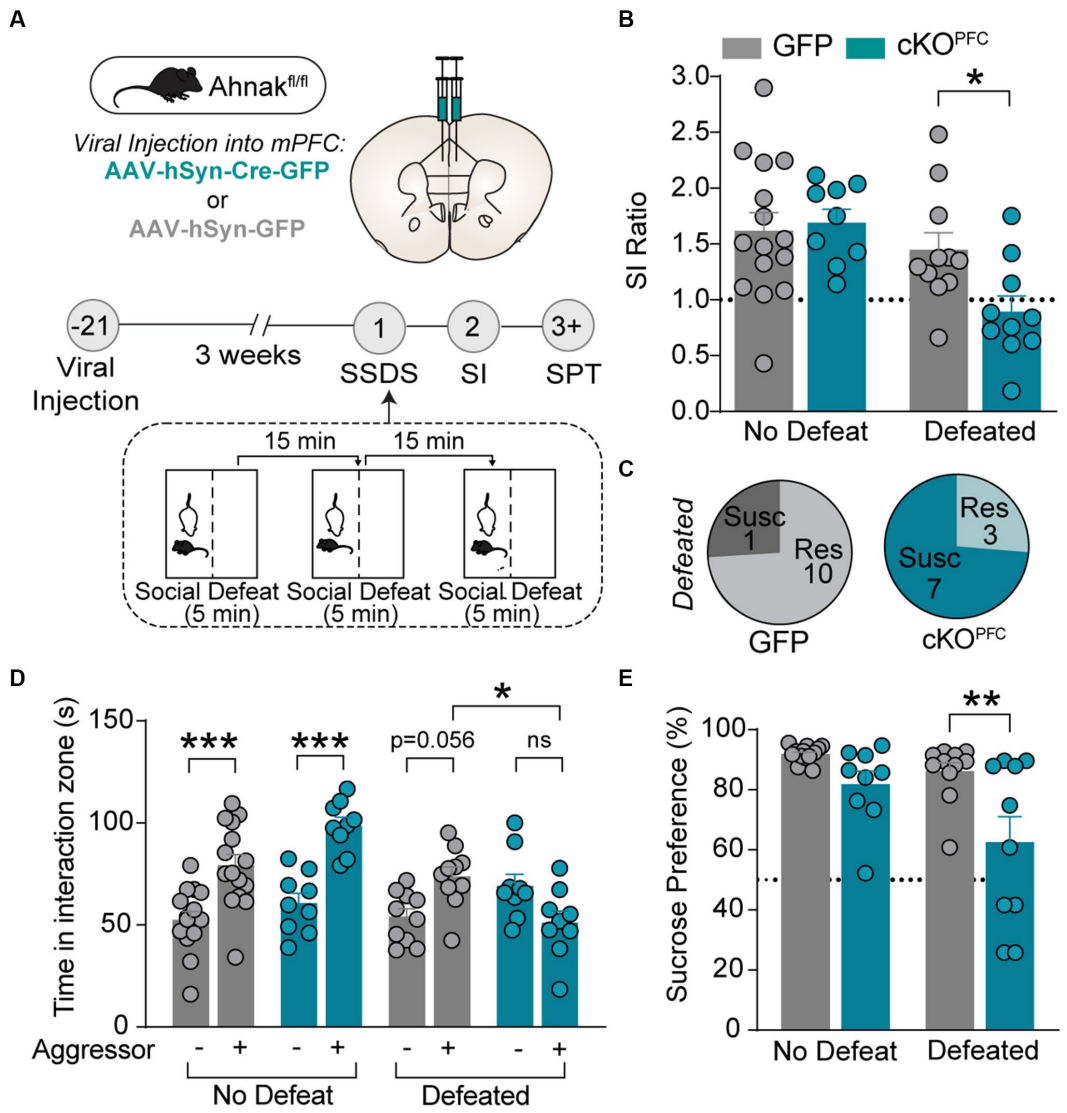
Ahnak in the PFC is required for behavioral resilience to stress. **(A)** Diagram and timeline of PFC-specific AAV-mediated KO approach, subthreshold social defeat stress (SSDS), and behavioral tests. **(B–E)** Ahnak cKO^PFC^ mice display a lower SI ratio **(B)**, a high ratio of susceptible mice **(C)**, a lower interaction time **(D)**, and a lower sucrose consumption compared with the levels of controls **(E)**. Non-defeat: GFP (*n* = 15) and cKO^PFC^ (*n* = 9), Defeated: GFP (*n* = 10–11) and cKO^PFC^ (*n* = 9–10). Two-way ANOVA, *Post-hoc* Bonferroni’s multiple comparisons. *p < 0.05, **p < 0.01, and ***p < 0.001, and ns, non-significant.

We also employed subthreshold social defeat stress (SSDS), a submaximal stressor, which does not evoke social avoidance in wild type (WT) mice but has been used to assess stress-susceptible factors (Krishnan et al., 2007; Golden et al., 2011; Cheng et al., 2019). Ahnak cKO^PFC^ and AAV-GFP control mice were subjected to SSDS, and subsequently social interaction and sucrose preference were measured (Figure 2A). SSDS exposure induces social avoidance as measured by decreased SI ratio and time and increases the proportion of susceptible mice in Ahnak cKO^PFC^ mice, while littermate controls maintained social interaction with novel aggressors (Figures 2B–D). Additionally, defeated Ahnak cKO^PFC^ mice display anhedonic-like behavior as measured by decreased sucrose consumption compared with defeated control mice (Figure 2E). However, non-defeated Ahnak cKO^PFC^ mice and control mice display comparable behaviors in SI test (Figures 2B,D) and SPT (Figure 2E). These results suggest that Ahnak is required for maintaining adaptive resilience to low-intensity stressors since its deletion results in increased behavioral measures of susceptibility such as social avoidance and anhedonic-like behavior. Across nearly all the experiments, there was no significant difference in locomotor activity between control and Ahnak cKO^PFC^ groups during social interaction test or in open field test (Supplementary Figures S1E–G). This is consistent with no difference in locomotor activity between WT and Ahnak null mice and Ahnak cKO^EMX^ mice (Jin et al., 2020).

**Figure 3:**
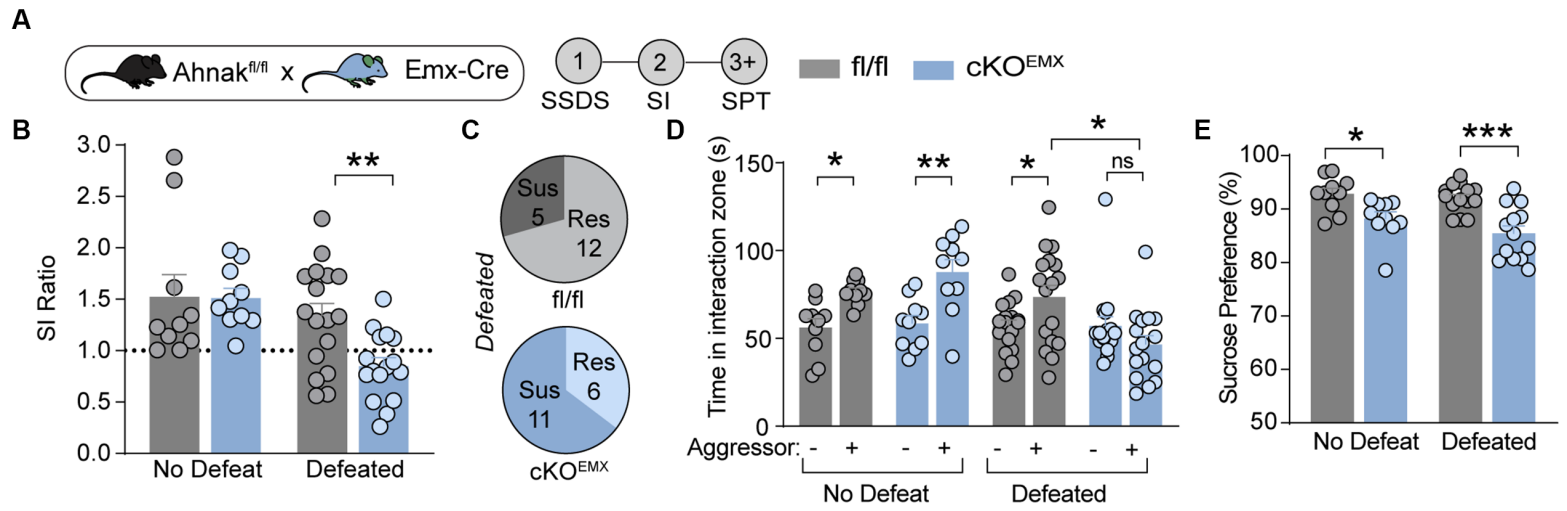
Ahnak in forebrain glutamatergic neurons is required for behavioral resilience to stress. **(A)** Diagram and timeline for SSDS and behavioral tests using floxed Ahnak mice crossed with EMX-Cre mice. **(B–E)** After SSDS, cKO^EMX^ mice (f/f; Cre/+) and control mice (f/f; Cre-negative: +/+) display a lower SI ratio compared with control mice (fl/fl) **(B)**. Pie charts showing the number of resilient and susceptible animals resulting after SSDS **(C)**. Defeated Ahnak cKO^EMX^ mice displaying a lower interaction time **(D)** and a lower sucrose consumption **(E)** compared with the levels of defeated control mice (fl/fl). Non-defeat: fl/fl (*n* = 9–10) and cKO^EMX^ (*n* = 10), Defeated: fl/fl (*n* = 17) and cKO^EMX^ (*n* = 13–17). Two-way ANOVA, *post-hoc* Bonferroni’s multiple comparisons. *p < 0.05, **p < 0.01, and ***p < 0.001, and ns, non-significant.

In our previous study, we observed baseline anhedonic-like and passive coping behaviors in mice with Ahnak deletion in *Emx1-*expressing forebrain glutamatergic neurons (cKO^EMX^) compared with floxed (fl/fl) littermate controls (Jin et al., 2020). To assess whether Ahnak cKO^EMX^ mice also display behavioral stress-susceptibility, we exposed cKO^EMX^ and control mice to SSDS, and subsequently, behavioral tests, SI and SPT, were performed (Figure 3A). Defeated Ahnak cKO^EMX^ mice showed decreased social preference, as measured by SI ratio and time interacting with a novel aggressor conspecific and an increased proportion of mice with behavioral-susceptibility compared with defeated control fl/fl mice (Figures 3B–D). Non-defeated Ahnak cKO^EMX^ mice and control mice display comparable behaviors in SI test (Figures 3B,C). However, both non-defeated and defeated Ahnak cKO^EMX^ mice show a decrease in sucrose consumption compared with controls in SPT (Figure 3E). The reduced sucrose consumption in non-defeated Ahnak cKO^EMX^ mice is due to their baseline depression-like behavior (Jin et al., 2020). The behavioral responses to social stress in Ahnak cKO^EMX^ mice are consistent with those found in Ahnak cKO^PFC^ mice (Figure 2). Altogether, our biochemical (Figure 1) and behavioral data derived from cell- and region-specific Ahnak knockout mice (Figures 2, 3) suggest an idea that Ahnak expressed in glutamatergic neurons in the PFC may play a critical role in behavioral resilience to social stress.

### Ahnak is required for the antidepressant effects of ketamine but not for fluoxetine, a selective serotonin reuptake inhibitor (SSRI)

**Figure 4:**
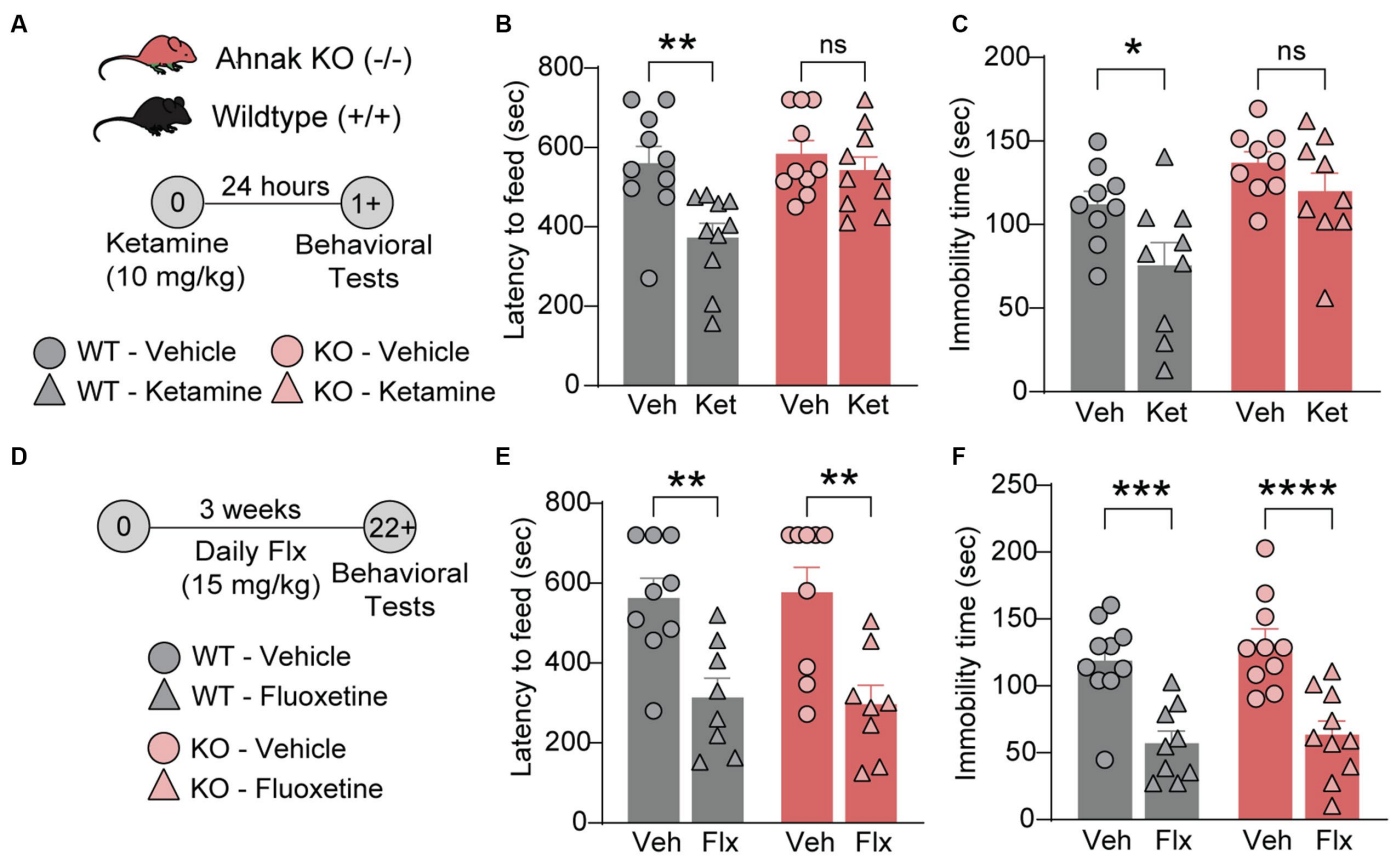
Ahnak is required for ketamine- but not fluoxetine-induced behavioral outcomes. **(A–C)** Diagram **(A)** for the behavioral effects of ketamine (10 mg/kg, i.p.) or vehicle in WT (+/+) and Ahnak null mice (−/−) in the NSF test **(B)** and FST **(C)**. **(D-F)** Diagram **(D)** for the behavioral effects of fluoxetine in the NSF test **(E)** and TST **(F)**. WT (+/+) and Ahnak null mice (−/−) were given fluoxetine (15 mg/kg, i.p., daily) or vehicle for 3 weeks. *n* = 9-10/group. *p < 0.05, **p < 0.01, ***p < 0.001, and ****p < 0.0001 and ns, non-significant. Two-way ANOVA with Bonferroni’s **(B, E, F)** or Newman–Keuls **(C)** *post-hoc* comparison.

Previous study has implicated p11, a key binding partner of Ahnak, in the actions of different classes of antidepressants including fluoxetine, a traditional SSRI antidepressant (Oh et al., 2013; Svenningsson et al., 2013), and ketamine, a fast-acting antidepressant (Sun et al., 2016). To determine whether Ahnak also mediates the behavioral effects of antidepressants, we examined the behavioral effects of ketamine and fluoxetine on WT and constitutive Ahnak KO mice. Both chronic fluoxetine and acute ketamine administrations result in a series of behavioral outcomes including decreased latency to feed in the novelty-suppressed feeding (NSF) test, a measure of suppressed hunger-induced exploration in a novel anxiogenic arena (Dulawa and Hen, 2005; Oh et al., 2013; Lee et al., 2015), and decreased immobility in the forced swim test (FST), a measure of engagement of passive coping strategies under threat (Commons et al., 2017). These behavioral outcomes are often used to measure antidepressant efficacy. We observed that the behavioral effects of ketamine were prevented in Ahnak KO mice in both the NSF test and FST (Figures 4A–C). However, chronic administration of fluoxetine similarly reduced both latency to feed and immobility time in WT and Ahnak KO mice (Figures 4D–F). While p11 together with its binding partner SMARCA3 mediate neurogenic and behavioral effects of SSRIs (Oh et al., 2013), our data suggest that Ahnak is dispensable in the actions of fluoxetine, but it is essential for the behavioral effects of the rapid antidepressant, ketamine. Together, these findings suggest that Ahnak-mediated mechanism for ketamine is distinct from the SMARCA3-mediated mechanism for SSRIs.

### Ahnak expression is associated with and required for the behavioral effects of ketamine in chronic social stress-exposed mice

**Figure 5:**
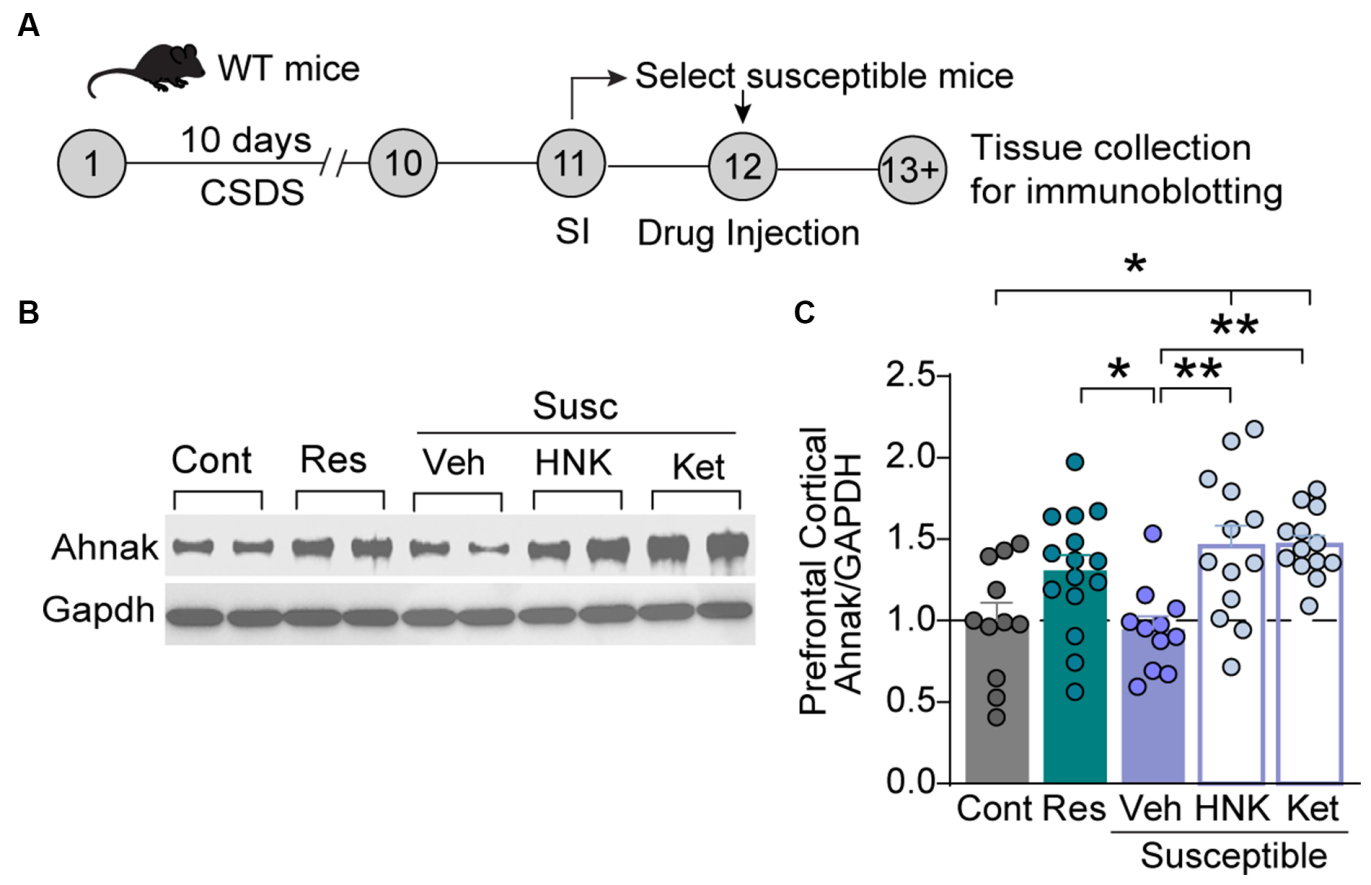
Ketamine or HNK administration increases PFC Ahnak expression to levels comparable to stress-resilient mice. **(A)** Timeline of CSDS and tissue collection. **(B)** Representative images of immunoblotting of Ahnak and Gapdh as a control. **(C)** Quantification of immunoblots. Non-defeat (Cont, *n* = 11 mice), resilient (Res, *n* = 15 mice), susceptible-vehicle-treated (Veh, *n* = 11 mice), susceptible-HNK-treated mice (HNK, *n* = 13 mice), and susceptible-ketamine-treated mice (Ket, *n* = 13 mice). *p < 0.05, **p < 0.01, one-way ANOVA with Bonferroni’s *post-hoc* comparison.

Despite Ketamine’s success for treatment-resistance depression (Krystal et al., 2020), the underlying mechanisms remain elusive. One key hypothesis underlying the restorative effects of fast-acting antidepressants is the enhancement of glutamatergic transmission in the PFC (Gerhard et al., 2020; Krystal et al., 2023). Indeed, ketamine enhances prefrontal plasticity via glutamatergic mechanisms (Moda-Sava et al., 2019), and the enhancement of excitatory neuron activity in the PFC is suggested as a critical mediator of antidepressant actions of ketamine (Abdallah et al., 2018; Wu et al., 2021). We, thus, sought to determine whether Ahnak may contribute to these underlying mechanisms due to its requirement for behavioral resilience via PFC and glutamatergic neuron mechanisms (Figures 2, 3). To assess the possibility of Ahnak as a molecular substrate of ketamine actions in the PFC, we first tested whether ketamine promotes Ahnak expression in susceptible mice to a level comparable to that of resilient mice. To do so, WT mice were subjected to CSDS, and then stress-susceptible mice were selected based on SI test scores. Saline (vehicle), ketamine (10 mg/kg, IP), or its active metabolite (*2R*, *6R*)-hydroxynorketamine (HNK) (10 mg/kg, IP) (Zanos et al., 2016) were injected to susceptible mice (Figure 5A). After 24 h, PFC tissues were collected for immunoblotting. Ahnak protein levels were significantly increased in the PFC of ketamine- or HNK-injected susceptible mice compared with the vehicle-injected susceptible mice (Figures 5B,C).

**Figure 6:**
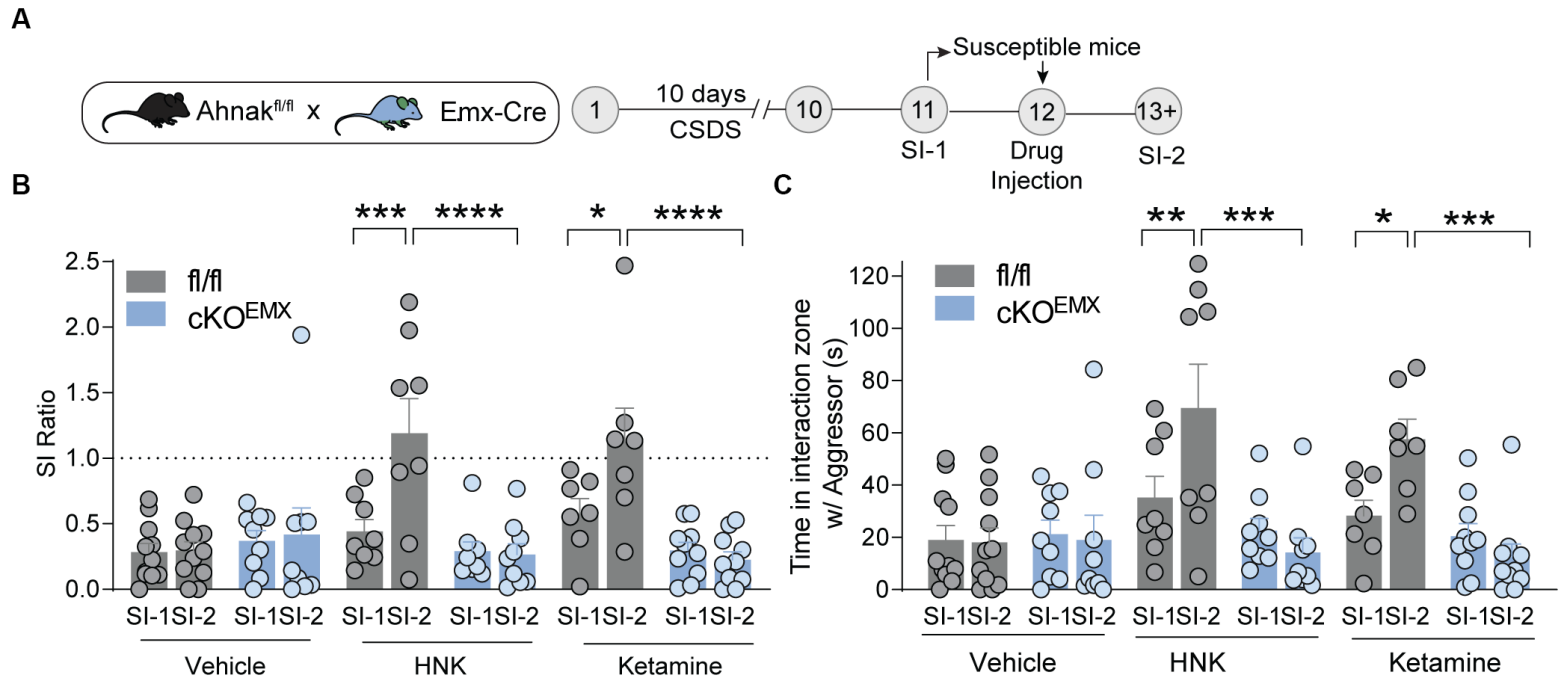
Ahnak in forebrain glutamatergic neurons is required for the restorative behavioral consequence of ketamine or HNK. **(A)** Diagram and timeline for CSDS, social interaction test before (SI-1) and after (SI-2) ketamine administration using floxed Ahnak mice crossed with EMX-Cre mice. **(B,C)** HNK or Ketamine administration increased SI ratio **(B)** or time interaction **(C)** in susceptible control (fl/fl) mice but not in susceptible Ahnak cKO^EMX^ mice. Susceptible-vehicle-treated (fl/fl, *n* = 11 mice; cKO^EMX^, *n* = 9), susceptible-HNK-treated mice (fl/fl, *n* = 8 mice; cKO^EMX^, *n* = 9), and susceptible-ketamine-treated mice (fl/fl, *n* = 7 mice; cKO^EMX^, *n* = 10). *p < 0.05, **p < 0.01, ***p < 0.001, and ****p < 0.0001, two-way ANOVA with Bonferroni’s *post-hoc* comparison.

To determine whether this increase in Ahnak expression in the PFC is required for ketamine-induced behavioral changes, we subjected Ahnak cKO^EMX^ and their littermate controls to CSDS. We again selected stress-susceptible mice based on SI ratio (SI-1) (Supplementary Figures S2A–C), administered drugs (vehicle, ketamine, or HNK), and conducted the SI test again (SI-2) (Figure 6A). Expectedly, ketamine and HNK increased SI ratio and time in fl/fl littermate controls, indicative of restoring ‘behavioral stress-resilience’ (Figures 6B,C). Ahnak deletion in the glutamatergic forebrain (cKO^EMX^ mice), however, abolished the behavioral effects of ketamine or HNK (Figures 6B,C), suggesting that Ahnak in glutamatergic forebrain neurons is required for antidepressant-like effects of ketamine or HNK. Locomotor activity between WT and Ahnak cKO^EMX^ mice was comparable during social interaction tests before and after drug administration (Supplementary Figures S2D,E). Altogether, these results suggest an essential role of Ahnak in glutamatergic neurons of the PFC in antidepressant-like behavioral effects of ketamine or its metabolite HNK.

### Ahnak deletion reduces glutamatergic transmission in the PFC

**Figure 7:**
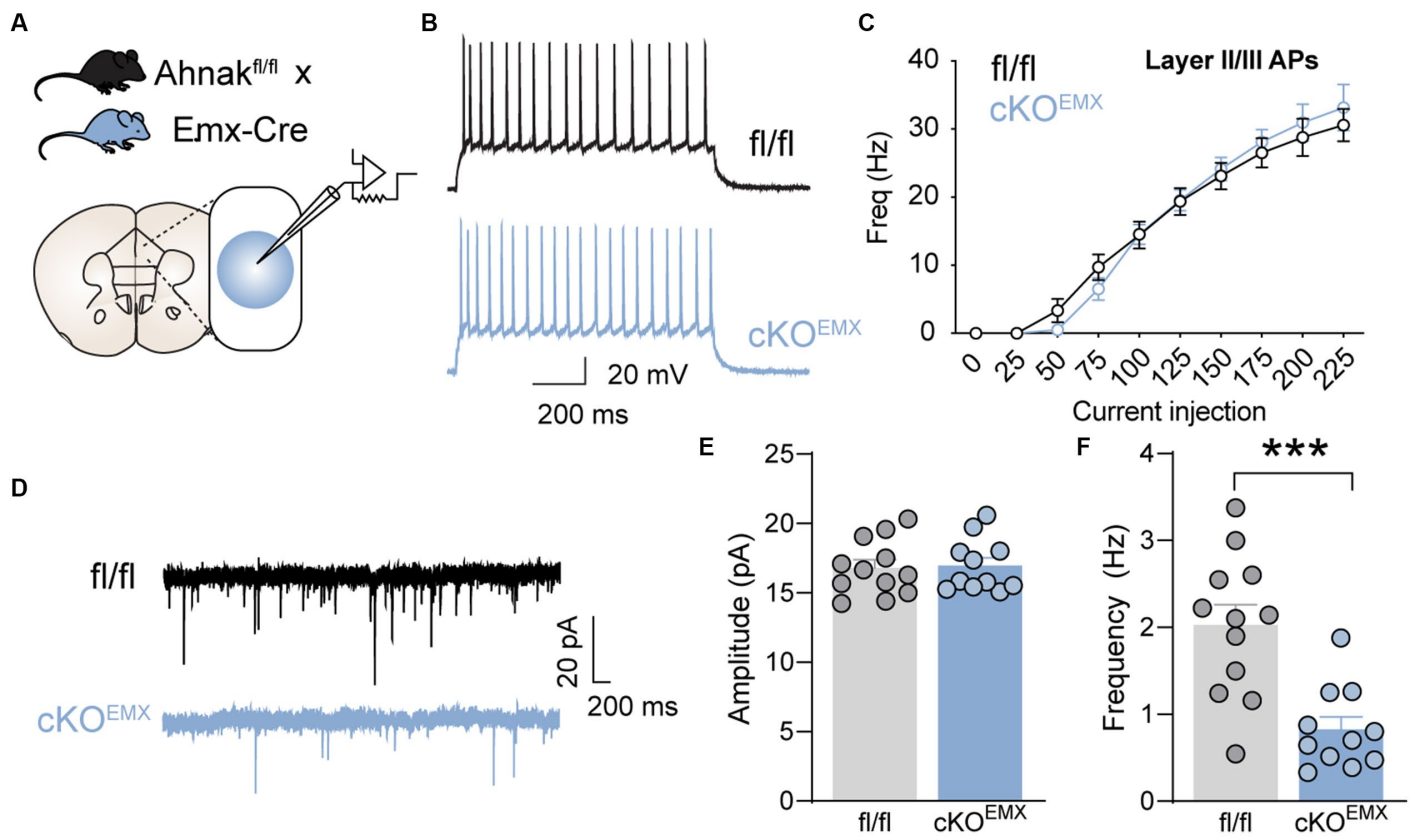
Forebrain glutamatergic neuron-specific Ahnak deletion decreases glutamatergic transmission in the layer II/III mPFC neurons. **(A)** Diagram of whole-cell patch clamp strategy for electrophysiological recordings of PFC layer II/III neurons in Ahnak cKO^EMX^ mice and controls. **(B)** A representative of action potential (AP) firing in the layer II/III cells in response to 150 pA injected current. Scale bar, 20 mV, 200 ms. **(C)** f-I plot showing AP frequency in layer II/III cells of control and EMX-Ahnak cKO^EMX^ mice. *n*= 9-10 cells /3 mice. **(D)** Representative traces of mEPSC. Scale bar, 20 pA, 200ms. **(E,F)** Bar graphs of amplitude **(E)** and frequency **(F)** of mEPSC. Student *t* test, ***p < 0.001. *n*= 11-12 cells / 3 mice.

One mechanism by which Ahnak may be critical for fast antidepressant action is through the modulation of glutamatergic transmission. We, thus, examined the effect of Ahnak deletion on glutamatergic transmission and excitability in the PFC. We used whole-cell patch-clamp and examined the electrophysiological properties in glutamatergic neurons in layer II/III of the PFC (Figure 7A). Layer II/III PFC neurons have been proposed to mediate depression-like or stress-induced behavioral abnormality in mice (Shrestha et al., 2015; Seo et al., 2017), and Ahnak and p11 are expressed in layer II/III (Seo et al., 2017; Jin et al., 2020). Ahnak was characterized as a regulator of L-type voltage-gated calcium channels, and ~ 50% of L-type calcium influx was reduced in the layer II/III pyramidal neurons in Ahnak cKO^EMX^ mice (Jin et al., 2020). Ahnak cKO^EMX^ did not alter the frequency of action potentials (Figures 7B,C), suggesting that neuronal excitability is unaffected by Ahnak deletion. However, we found that the frequency, but not the amplitude, of mini excitatory postsynaptic currents (mEPSCs) in glutamatergic neurons in layer II/III of the PFC of Ahnak cKO^EMX^ were markedly reduced compared with fl/fl controls (Figures 7D–F). Thus, Ahnak expression may contribute to behavioral resilience or behavioral effects of ketamine via the modulation of glutamatergic transmission.

## Discussion

In this study, we describe a molecular mechanism underlying behavioral resilience to CSDS. Using biochemical and transgenic approaches, we identify Ahnak expressed in forebrain excitatory neurons and the PFC as a regulator of stress-induced behavioral adaptations and a mediator of antidepressant-like effects of ketamine or its metabolite HNK. Furthermore, we find that deletion of Ahnak reduces glutamatergic transmission in PFC layer II/III neurons. Thus, our study suggests that cortical Ahnak in excitatory PFC neurons drives behavioral resilience in response to chronic social stress or behavioral recovery by fast-acting antidepressants in stress-susceptible subjects, at least in part, via modulation of glutamatergic transmission.

Ahnak alterations associated with stress-induced behavioral adaptations are divergent depending on cell types and brain regions. In earlier studies, we found that Ahnak expression in parvalbumin-expressing GABAergic interneurons in the ventral hippocampus was critical for stress-susceptibility. Lower expression of Ahnak was associated with behavioral resilience but higher expression was found in stress-susceptible mice (Bhatti et al., 2022). Interestingly, in this study, we have observed an opposing pattern of Ahnak expression in the PFC of stress-resilient mice (Figures 1B,C). Ahnak expression in the PFC and hippocampus is inversely correlated (Supplementary Figure S3). These opposing patterns are likely mediated by circuit and cellular activity in specific brain regions expressing Ahnak. Because divergent Ahnak expression is causally related to divergent behavioral adaptations, Ahnak holds the potential for being further developed as a biomarker of behavioral adaptations in response to chronic stress. However, the pattern of changes of p11 and Anxa2 is somewhat different from Ahnak changes in PFC and hippocampus. Although p11 protein levels were slightly but significantly higher in the PFC of resilient mice compared with the levels in non-defeated control mice; p11 expression in the PFC of mice was not correlated with time spent in the interaction zone containing an aggressive conspecific or SI ratio (Supplementary Figure S4A). Hippocampal p11 is reduced in stress-susceptible mice, and p11 expression in the hippocampus of mice was positively correlated with time spent in the interaction zone containing an aggressive conspecific but not with the SI ratio (Supplementary Figure S4B). In contrast, CSDS increases AnxA2 levels in both the PFC and hippocampus of stress-susceptible mice, resulting in inverse correlation between Ahnak levels and social interaction (Supplementary Figures S4C,D). We found correlated protein levels between Ahnak, p11, and Anxa2 in the hippocampus and prefrontal cortex in our published study (Jin et al., 2020) and in this study with non-stressed mice (Supplementary Figure S5A). However, after CSDS the positive correlations were maintained only for Ahnak-Anxa2 and p11-Anxa2 in the hippocampus, suggesting active molecular changes of the Ahnak/p11/Anxa2 complex in the CSDS condition (Supplementary Figure S5B).

Divergent behavioral outcomes of cell-type-specific Ahnak deletions suggest a pivotal role of Ahnak in the regulation of neuronal and circuit activities. Ahnak deletion in the ventral hippocampal PV neurons promotes behavioral resilience to CSDS, a phenotype that is recapitulated by chemogenetic inhibition of PV neurons (Bhatti et al., 2022). In contrast, deletion of Ahnak in forebrain glutamatergic neurons (cKO^EMX^) displays base line depression-like behavior (Jin et al., 2020). In this study, we show that both Ahnak cKO^EMX^ and cKO^PFC^ confer stress-susceptibility (Figures 2, 3). In the PFC, Ahnak deletion reduces glutamatergic transmission to layer II/III prelimbic cortex neurons (Figure 7). Given these results, it is likely that the effects of Ahnak deletion on glutamatergic transmission in PFC dominantly contribute to anhedonic-like, and passive coping behavior observed in constitutive Ahnak deletion in the entire body (Jin et al., 2020). Indeed, neuronal activity or excitatory synaptic efficacy in PFC is reduced in depressed patients and stressed mice (Kang et al., 2012; Thompson et al., 2015; Howard et al., 2019), and optogenetic activation of PFC promotes behavioral resilience (Covington 3rd et al., 2010), supporting the interpretation of our findings.

Ketamine is an open channel blocker of ionotropic glutamatergic N-Methyl-D-Aspartate (NMDA) receptors. Animal studies with subanesthetic doses of ketamine have identified key mechanisms underlying antidepressant actions of ketamine. The inhibition of NMDA receptors by ketamine on inhibitory interneurons and disinhibition of glutamate release have been suggested as one of the major mechanisms underlying antidepressant actions of ketamine (Krystal et al., 2023). In this study, we have observed that Ahnak cKO^EMX^ significantly reduces the frequency of AMPAR currents in pyramidal neurons in the layer II/III of PFC (Figure 7). Previous studies have determined that chronic stress reduces glutamatergic transmission in PFC (Seo et al., 2017; Hughes et al., 2023), while fast-acting antidepressants increase glutamatergic synapse plasticity (Moda-Sava et al., 2019; Wu et al., 2021). Although we did not assess evoked glutamate-mediated currents or mEPSCs in CSDS-exposed mice or under the condition of ketamine administration, we posit that Ahnak deletion would suppress ketamine-induced plasticity, whereas its overexpression may promote glutamatergic transmission and thus behavioral resilience.

p11 pathways have been implicated in the behavioral effects of antidepressants (Svenningsson et al., 2006; Oh et al., 2013; Svenningsson et al., 2013), and p11 changes might be developed as biomarkers of antidepressant efficacy of therapeutic approaches (Svenningsson et al., 2014; Neyazi et al., 2018). p11 levels are increased in the brains of mice treated with electroconvulsive therapy or administered with imipramine or selective serotonin reuptake inhibitors (SSRIs), such as fluoxetine or citalopram, and p11 KO mice abolished the behavioral effects of SSRIs (Svenningsson et al., 2006; Oh et al., 2013; Svenningsson et al., 2013). p11 is also increased by ketamine, and p11 knockdown diminished the fast-acting antidepressant-like actions of ketamine in mice (Sun et al., 2016). Studies of p11-binding partners have contributed to the understanding of neurobiology of p11 and revealed mechanisms underlying antidepressant actions in mice (Oh et al., 2013; Lee et al., 2015; Chen et al., 2022). In our previous study, Ahnak protein was identified as a major scaffolder and stabilizer of p11 and annexin A2 proteins in the brain (Jin et al., 2020). The baseline expression patterns of p11 and Ahnak in the hippocampus and PFC in individual animals are highly correlated with each other (Jin et al., 2020). Both constitutive Ahnak KO mice and p11 KO mice display depression-like behavior (Svenningsson et al., 2006; Jin et al., 2020). In a restraint stress paradigm, a reduction in p11 expression was found in the PFC layer II/III neurons (Seo et al., 2017). Both stress-induced depression-like behaviors and p11 expression are reversed by antidepressants such as SSRIs. In stressed animals, the overexpression of p11 rescues depression-like behaviors by restoring glutamatergic transmission (Seo et al., 2017). Although p11 and Ahnak function have not been parallelly compared with the restraint stress experiments or CSDS study, their alteration patterns and function in cellular and behavioral responses to chronic stress appear similar.

In this study, we also show that Ahnak is dispensable in behavioral effects of fluoxetine (Figures 4D–F). Our previous study indicates that another p11-binding partner SMARCA3, a chromatin remodeling factor, mediates neurogenic and behavioral effects of SSRIs (Oh et al., 2013). In contrast, Ahnak is required for antidepressant-like effects of ketamine and its metabolite HNK (Figures 4A–C). Ahnak expression is increased in ketamine- or HNK-administered mice, and Ahnak cKO^EMX^ abolished the beneficial effects of ketamine or HNK in the CSDS paradigm (Figures 5, 6). Because the SMARCA3 pathway is not altered by the administration of ketamine (Umschweif et al., 2021), the bifurcated p11 pathways mediated by SMARCA3 or Ahnak are likely involved in the antidepressant-like actions of SSRI or ketamine, respectively.

In addition to the p11/annexin A2 complex, Ahnak scaffolds L-type-VGCCs (Jin et al., 2020) and S100B (Gentil et al., 2001). p11 (S100a10) and S100B are members of S100 family proteins and EF-hand Ca^2+^-binding protein superfamily (Bhattacharya et al., 2004; Ikura and Ames, 2006), many of which undergo large conformational change upon binding to Ca^2+^. Although two EF-hand motifs in p11 are mutated, the p11/annexin2 complex is responsive to Ca^2+^ via annexin A2, which binds to Ca^2+^ (Bharadwaj et al., 2013). While S100B interacts with repeated motifs in the central region of Ahnak (Gentil et al., 2001), the N-terminal region of Ahnak interacts with a pore-forming α1 subunit (Cav1.2 or Cav1.3) of L-type calcium channels, but C-terminal region interacts with auxiliary β subunits of calcium channels and p11/annexin A2 complex (Oh et al., 2013; Jin et al., 2020). Ahnak regulates the cell surface localization of L-type calcium channels and regulates L-type-specific calcium influx in neurons (Jin et al., 2020). These results altogether strongly suggest that Ahnak is a mediator of calcium signaling in neurons. However, whether Ahnak-mediated calcium signaling is involved in glutamatergic transmission remains to be studied. Previous studies of Ahnak expression in the brain using immunohistochemistry revealed its expression not only in neurons but also in non-neuronal cells including endothelial cells in the blood–brain barrier and epithelial cells in the choroid plexus (Gentil et al., 2005; Jin et al., 2020). Because alterations of p11 (Svenningsson et al., 2006, 2013; Chen et al., 2022), S100B (Schroeter and Steiner, 2009; Steiner et al., 2009), and L-type VGCCs (Heyes et al., 2015; Nanou and Catterall, 2018) are implicated in the pathophysiology of psychiatric disorders, further studies of upstream regulators and downstream pathways of Ahnak and their function in specific brain cell types would accelerate our understanding of neurobiology and pathogenic mechanisms underlying psychiatric disorders.

## Data availability statement

The raw data supporting the conclusions of this article will be made available by the authors, without undue reservation.

## Ethics statement

The animal study was approved by the Rockefeller University Institutional Animal Care and Use Committee. The study was conducted in accordance with the local legislation and institutional requirements.

## Author contributions

DB: Conceptualization, Writing – review & editing, Investigation, Formal analysis. JJ: Writing – review & editing, Formal analysis, Investigation. JC: Formal analysis, Investigation, Writing – review & editing. KM: Investigation, Writing – review & editing. K-WL: Investigation, Writing – review & editing. CB: Formal analysis, Writing – review & editing. YJ: Formal analysis, Investigation, Writing – review & editing. SS: Resources, Writing – review & editing. YK: Conceptualization, Funding acquisition, Resources, Supervision, Writing – original draft, Writing – review & editing, Formal analysis.
