# Supplementary material for: Ahnak in the prefrontal cortex mediates behavioral correlates of stress resilience and rapid antidepressant action in mice
Source: Front Mol Neurosci. 2024 May 17;17:1350716. doi: 10.3389/fnmol.2024.1350716 (PMC11140847; doi:10.3389/fnmol.2024.1350716)
Supplement: Supplementary file 1 [file Data_Sheet_1.docx]

**Supplementary Figures**

**
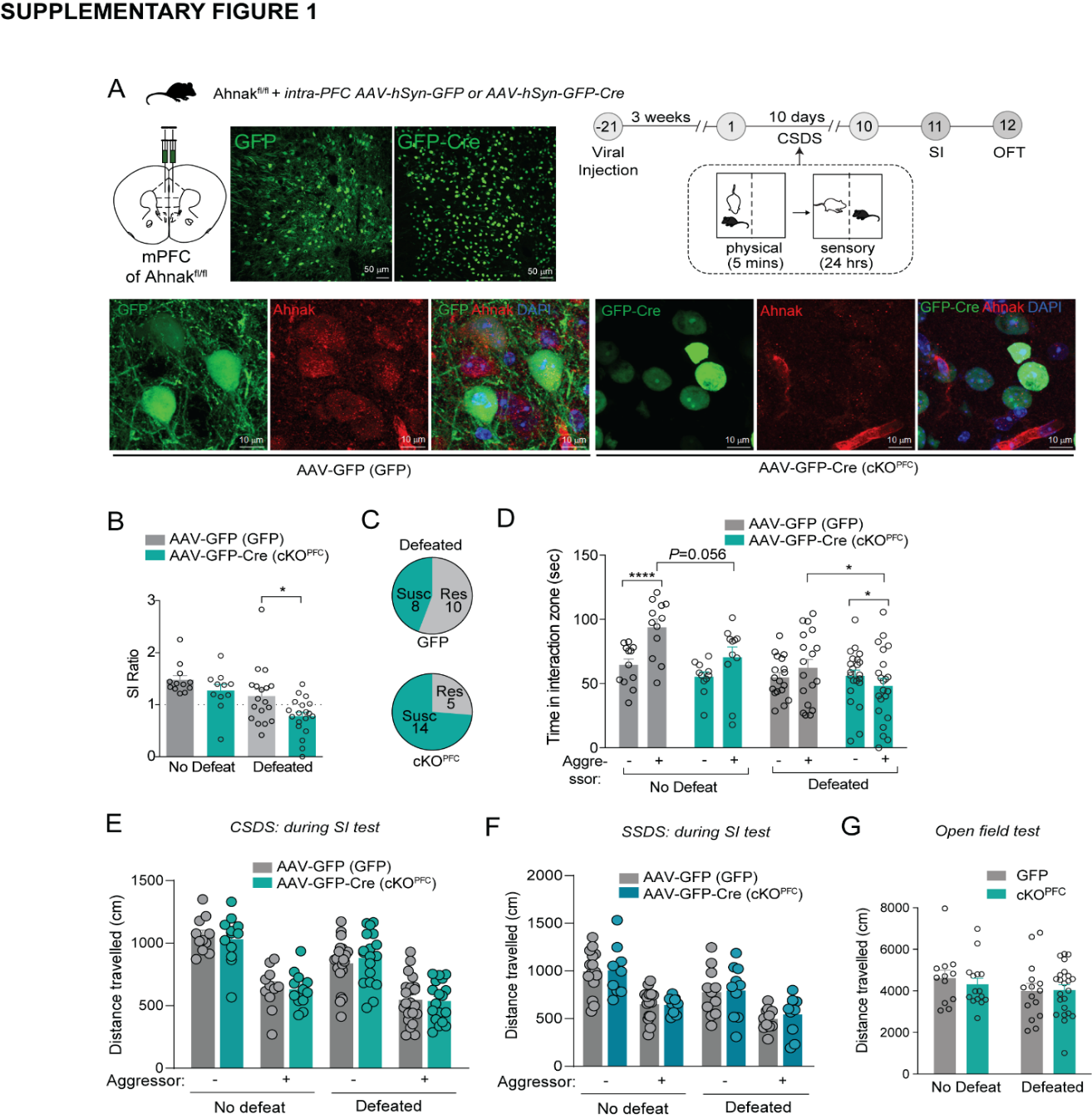
**

**Supplementary Figure 1. PFC-specific deletion of Ahnak increases behavioral susceptibility to CSDS.** (**A**) Diagram and timeline for PFC-specific knockout approach using stereotaxic AAV injections of AAV-Cre-GFP (cKO^PFC^) or AAV-GFP control (GFP), chronic social defeat stress (CSDS) and social interaction test (SI). Representative images of GFP or GFP-Cre expression in the PFC and Ahnak are shown. (**B-D**) Ahnak cKO^PFC^ mice display a lower SI ratio (**B**) and a large proportion of susceptible mice among defeated mice (**C**) and a lower interaction time (**D**) compared to the levels of GFP control mice. Non-defeat: GFP (n=12) and cKO^PFC^ (n=11), Defeated: GFP(n=18) and cKO^PFC^ (n=22). (**E, F**) Locomotor activity during social interaction test. (**G**) Total distance traveled for control mice and cKO^PFC^ mice in open field test. Two-way ANOVA, *Post-hoc* Bonferroni’s multiple comparisons. **p*<0.05 and *****p* < 0.0001.

**
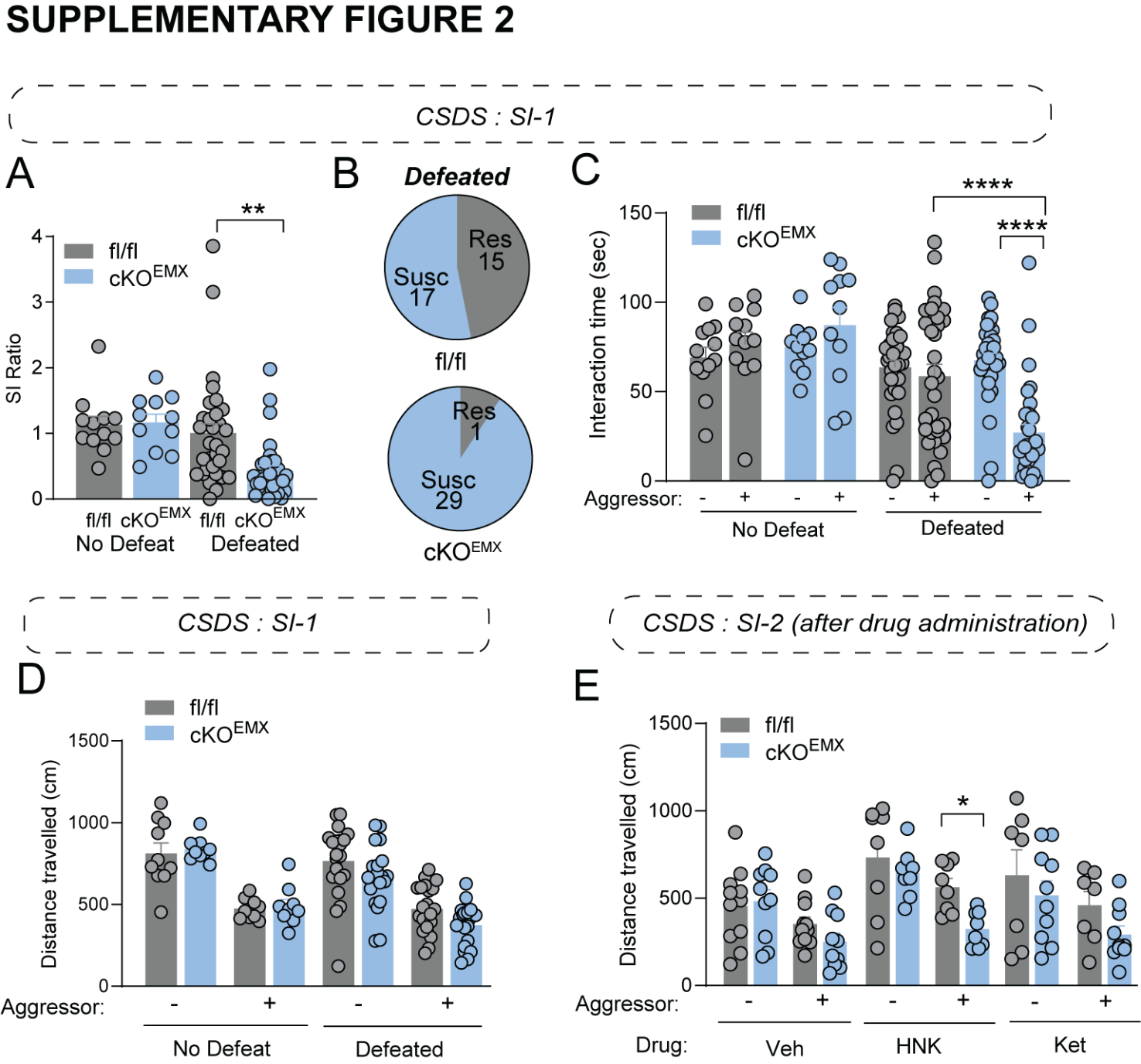
**

**Supplementary Figure 2. Ahnak deletion in glutamatergic forebrain neurons exacerbates stress-susceptibility after CSDS.** (**A-C**) After CSDS, Ahnak cKO^EMX^ mice display a lower SI ratio (**A**), a high ratio of susceptible mice (**B**), and a lower interaction time compared to the levels of controls during SI-1 before drug administration (**C**). Non-defeat: fl/fl (n=12) and cKO^EMX^ (n=11), Defeated: fl/fl (n=32) and cKO^EMX^ (n=32). (**D, E**) Distance travelled during SI-1 (**D**) and SI-2 (**E**) tests for control (fl/fl) and Ahnak cKO^EMX^ mice. Non-defeat: fl/fl (n=10) and cKO^EMX^ (n=9), Defeated: fl/fl (n=22) and cKO^EMX^ (n=21). Two-way ANOVA, *Post-hoc* Bonferroni’s multiple comparisons. **p*<0.05, ***p* < 0.01, ****p*<0.001, and ns, nonsignificant.


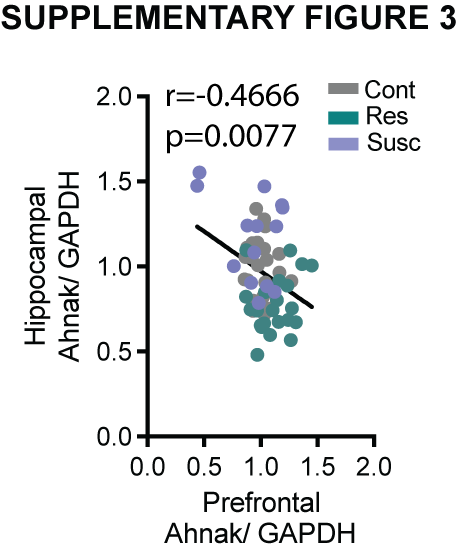


**Supplementary Figure 3. Ahnak expression in the hippocampus and PFC are negatively correlated.** Ahnak protein levels in the hippocampus are inversely correlated with the levels in the PFC of the mice exposed to CSDS (pearson *r* : *r* = 0.4666, *p*=0.0077, n=62 mice). All data were normalized to Cont (no defeat) group.

**
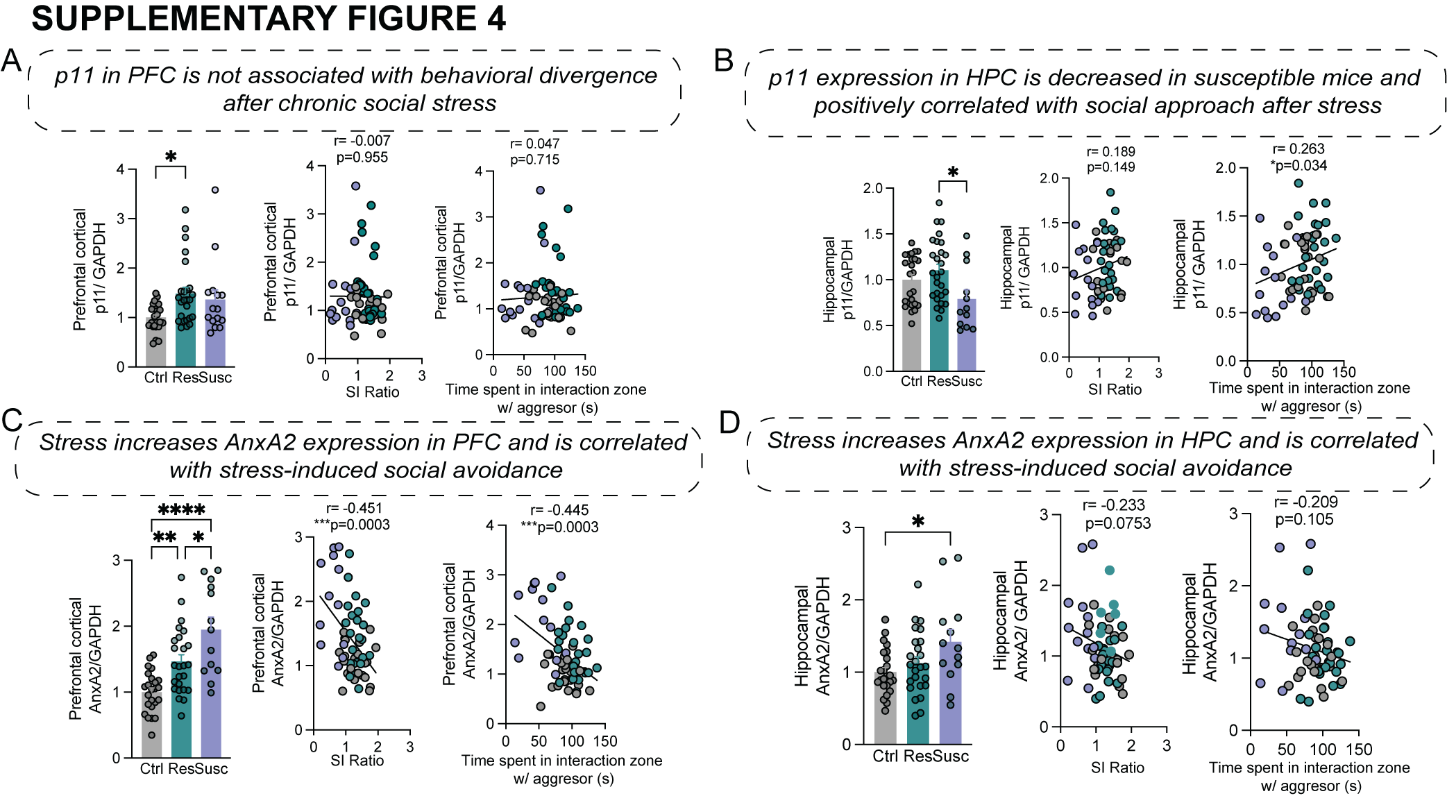
**

**Supplementary Figure 4. p11 and AnxA2 expression in PFC and hippocampus after chronic social stress. (A)** *Left,* Prefrontal p11 expression is increased in resilient mice (n=15-23/group). *Middle and Right,* Prefrontal p11 expression does not correlate with behavioral correlates of stress-resilience as measured by SI ratio (pearson *r* = -0.007, *p=*0.955) or time in interaction zone with aggressor (pearson *r* = 0.047, *p=*0.715). **(B)** *Left,* Hippocampal p11 expression is decreased in susceptible mice compared to resilient (n=12-27/group). *Middle and Right,* Hippocampal p11 expression does not significantly correlate with SI ratio (pearson *r* = 0.189, *p=*0.149) but is slightly correlated with time in interaction zone with aggressor (pearson *r* = 0.263, *p=*0.034). **(C)** *Left,* Prefrontal AnxA2 expression is increased in after chronic social stress (n=13-23/group). *Middle and Right,* Prefrontal AnxA2 is negatively correlated with behavioral correlates of stress-resilience as measured by SI ratio (pearson *r* = -0.451, *p=*0.0003) or time in interaction zone with aggressor (pearson *r* = -0.445, *p=*0.0003). **(D)** *Left,* Hippocampal AnxA2 expression is increased in stress-susceptible mice (n=13-25/group). *Middle and Right,* Prefrontal AnxA2, however, is not significantly associated with behavioral correlates of stress-resilience as measured by SI ratio (pearson *r* = -0.233, *p=*0.0753) or time in interaction zone with aggressor (pearson *r* = -0.209, *p=*0.105).

**
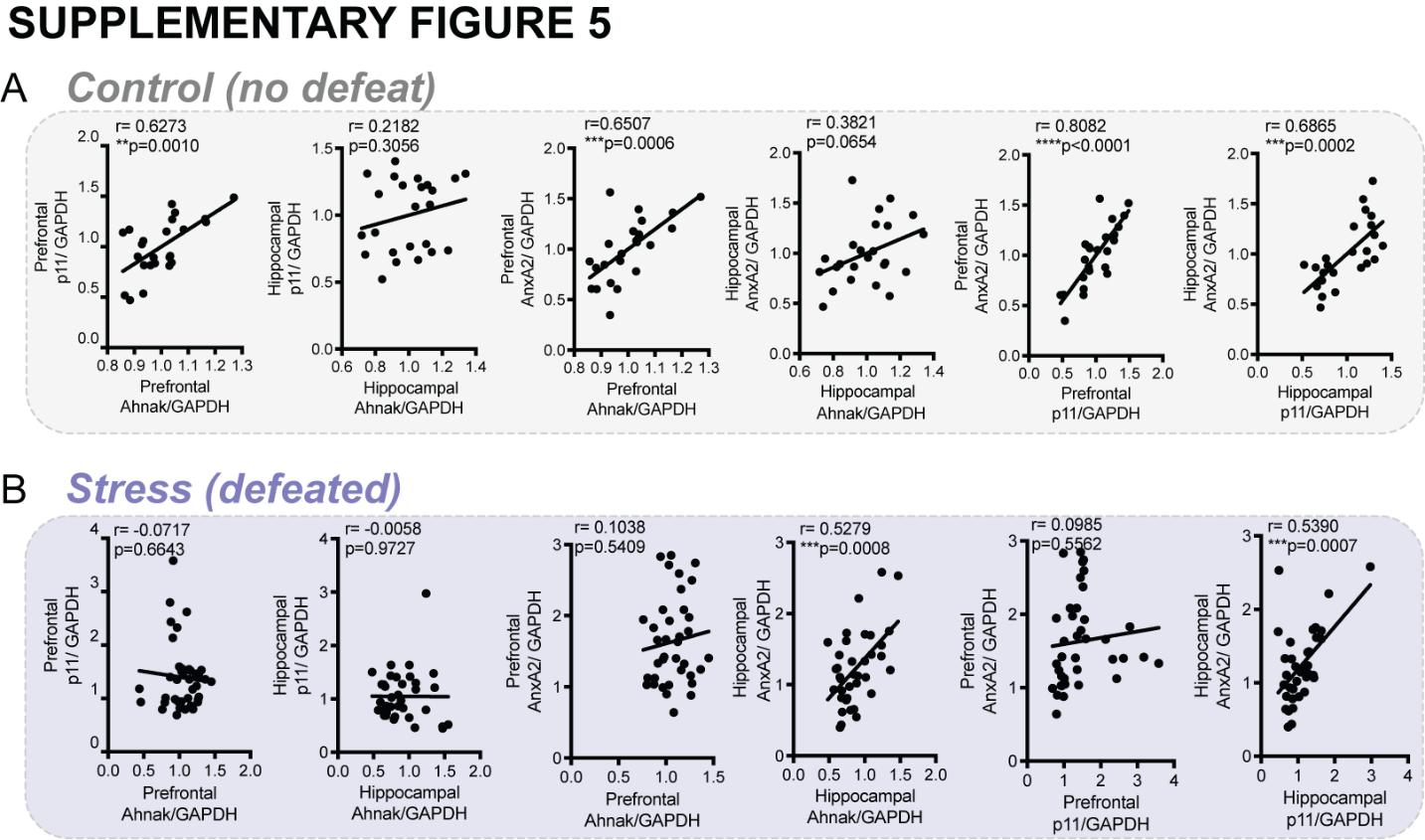
**

**Supplementary Figure 5. Stress disrupts the correlations between the protein levels of p11, AnxA2 and Ahnak.** Pearson’s correlation analysis comparing within-sample expression of prefrontal or hippocampal Ahnak, p11, and AnxA2 in (**A**) control (n=23 mice) and (**B**) CSDS-exposed mice (n=36-38 mice). CSDS blunts correlations between prefrontal Ahnak, p11 and AnxA2 proteins, as well as hippocampal correlation between Ahnak and p11. Hippocampal correlation between Ahnak and Anxa2 or p11 and Anxa2 remains intact after exposure to stress.
